# Supplementary material for: Morphology of an Early Oligocene beaver Propalaeocastor irtyshensis and the status of the genus Propalaeocastor
Source: PeerJ. 2017 May 16;5:e3311. doi: 10.7717/peerj.3311 (PMC5436589; doi:10.7717/peerj.3311)
Supplement: Supplemental Information 2 [file peerj-05-3311-s002.docx]

Barbour EH, Schultz CB. 1937. An early Pleistocene fauna from Nebraska. *American Museum Novitates* 942:1-10.

Bendukidze OG, De Bruijn H, Van Den Hoek Ostende LW. 2009. A revision of Late Oligocene associations of small mammals from the Aral Formation (Kazakhstan) in the National Museum of Georgia, Tbilissi. *Palaeodiversity* 2:343-377.

Borisoglebskaya MB. 1967. A new genus of beavers from the Oligocene of Kazakhstan. *Byulleten Moskovskogo Obshchestva Ispytaleley Prirody, Otdel Biologicheskiy* 72:129-135.

Casanovas-Vilar I, Alba DM, Almécija S, Robles J, Galindo J, Moyà-Solà S. 2008. Taxonomy and paleobiology of the genus Chalicomys Kaup, 1832 (Rodentia, Castoridae), with the description of a new species from Abocador de Can Mata (Vallès-Penedès Basin, Catalonia, Spain). *Journal of Vertebrate Paleontolgoy* 28:851-862.

Chow MC, Li CK. A correction of the age of the hsiatsaohwan Formation and its mammalian fauna. *Acta Stratigrphica Sinica* 2:122-130

Emry RJ. 1972. A new species of *Agnotocasto*r (Rodentia, Castoridae) from the Early Oligocene of Wyoming. *American Museum Novitates* 2485:1-7.

Hugueney M. 1975. Les Castoridae (Mammalia, Rodentia) dans l'Oligocène d'Europe. *Colloque internationaux du Centre national de la recherche scientifique* 218:791-804.

Hugueney M. 1999. Family Castoridae. In: Rössner GE, and Heissig K, eds. *The Miocene Land Mammals of Europe*. München: Verlag Dr. Friedrich Pfeil, 281-300.

Korth WW. 1988. A new species of beaver (Rodentia, Castoridae) from the middle Oligocene (Orellan) of Nebraska. *Journal of Paleontology* 62:965-967.

Korth WW. 1998. A new beaver (Rodentia, Castoridae) from the Orellan (Oligocene) of North Dakota. *Paludicola* 1:127-131.

Korth WW. 1996a. Additional specimens of *Agnotocastor readingi* (Rodentia, Castoridae) from the Orellan (Oligocene) of Nebraska and a possible origin of the beavers. *Paludicola* 1:16-20.

Korth WW. 1996b. A new genus of beaver (Mammlia: Castoridae: Rodentia) from the Arikareean (Oligocene) of Montana and its bearing on castorid phylogeny. *Annals of Carnegie Museum* 65:167-179

Korth WW. Topotypic cranial material of the beaver Monosaulax pansus Cope (Rodentia, Castoridae). *Paludicola* 4:1-5.

Korth WW, Rybczynski N. 2003. A new, unusual castorid (Rodentia) from the earliest Miocene of Nebraska. *Journal of Vertebrae Paleontology* 23:667-675.

Korth WW. 2004. Beavers (Rodentia, Castoridae) from the Runningwater Formation (Early Miocene, Early Hemingfordian) of western Nebraska. *Annals of Carnegie Museum* 73:1-11.

Korth WW, Samuels JX. 2015. New rodent material from the John Day Formation (Arikareean, middle Oligocene to early Miocene) of Oregon. *Annals of Carnegie Museum* 83:19-84.

Lopatin AV. 2003. The revision of the Early Miocene beavers (Castoridae, Rodentia, Mammalia) from the North Aral region. *Russian Journal of Theriology* 2:15-25.

Lopatin AV. 2004. Early Miocene small mammals from the north Aral region (Kazakhstan) with special reference to their biostratigraphic significance. *Paleontological Journal* 38:S217-S323.

Lytschev GF. 1970. New species of beaver from the Oligocene of the northern Aral region. *Paleontologicheskia Zhurnal* 1970:84-89.

Lytschev GF. 1978. A new early Oligocene beaver of the genus *Agnotocastor* from Kazakhstan. *Paleontologicheskia Zhurnal* 12:128-130.

Lytschev GF, Shevyreva NS. 1994. Beavers (Castoridae, Rodentia, Mammalia) from Middle Oligocene of Zaissan Depression (Eastern Kazakhstan). In: Vangengeim EA, Pevzner MA, and Tesakov AS, eds. *Paleoteriologiya, Voprosi Teriologii*. Moscow: Nauka, 79-106.

MacDonald JR. 1963. The Miocene faunas from the Wounded Knee area of western South Dakota. *Bulletin of the American Museums of Natural History* 125: 143-238.

Misonne X. 1957. Mammifères Oligocènes de Hoogbutsel et Hoeleden: I. Rongeurs et Ongulés. *Bulletin de l’Institut Royal des Sciences Naturelles de Belgique* 33:1-16.

Mörs T, Stefen C. 2010. The castorid *Steneofiber* from NW Germany and its implications for the taxonomy of Miocene beavers. *Acta Palaeontologica Polonica* 55:189-198.

Mörs T, Tomida Y, Kalthoff DC. 2016. A new large beaver (Mammalia, Castoridae) from the early Miocene of Japan. *Journal of Vertebrate Paleontology* e1080720. DOI: 10.1080/02724634.2016.1080720

Peterson OA. 1905. Description of new rodents and discussion of the origin of *Daemonelix*. *Memoirs of the Carnegie Museum* 2:139-202.

Prieto J, Casanovas-Vilar I, Gross M. 2014. *Euroxenomys* *minutus minutus* (Rodentia, Castoridae) from Gratkorn (Austria, Styria). *Palaeobio Palaeoenv* 94:163-170.

Qiu ZD. 1996. *Middle Miocene Micromammalian Fauna from Tunggur*, *Nei Mongol*. Beijing: Science Press.

Qiu ZD, Li Q. 2016. Neogene rodents from central Nei Mongol, China. *Palaeontologia Sinica, New Series C* 30:1-684

Rybczynski, N, Ross EM, Samuels JX, Korth WW. 2010. Re-evalutation of *Sinocastor* (Rodentia: Castoridae) with implications on the origin of modern beavers. PLoS ONE 5:e13990. DOI:10.1371/journal.pone.0013990.

Stefen C. 1997. *Steneofiber eseri* (Castoridae, Mammalia) von der Westtagente bei Ulm im Vergleich zu anderen Biberpopulationen. *Stuttgarter Beiträge zur Naturkunde serie B (Geologie und Palaöntologie)* 255:1-73.

Stefen C. 2014. Cranial morphology of the Oligocene beaver Capacikala gradatus from the John Day Basin and comments on the genus. *Palaeontologia Electonica* 17:1-29.

Stirton RA. 1934. A new species of Amblycastor from the Platybelodon Beds, Tung Gur Formation, of Mongolia. *American Museum Novitates* 694:1-4.

Stirton RA. 1935. A review of the Tertiary beavers. *University of California Publications Bulletin of the Department of Geological Sciences* 23:391-458.

Wilson RW. 1949b. On some White River fossil rodents. *Carneigie Institution of Washington Publication* 584:27-50.

Wood AE. 1974. Early Tertiary vertebrate faunas Vieja Group Trans-Pecos Texas: Rodentia. *Texas Memorial Museum Bulletin* 21:1-112

Wu W, Meng J, Ye J, Ni X. 2004. *Propalaeocastor* (Rodentia, Mammalia) from the early Oligocene of Burqin Basin, Xinjiang. *American Museum Novitates* 3461:1-16.

Young CC. 1955. On a new Trogontherium from Hsiatsaohwan, Shihunghsien and with notes on the mammalian remians from Chi-tsu Wuhohsien, Anhwei. *Acta Palaeontoloica Sinica* 3:64-67.

Xu XF. 1994. Evolution of Chinese Castoridae. In: Tomida Y, Li CK, Setoguchi T (eds.) Rodent and Lagomorph Families of Asian Origins and Diversification. *National Science Museum Monographs* 8:77-95.
